# EHealth Literacy in UK Teenagers and Young Adults: Exploration of Predictors and Factor Structure of the eHealth Literacy Scale (eHEALS)

**DOI:** 10.2196/14450

**Published:** 2020-09-08

**Authors:** Patricia Holch, Jordan R Marwood

**Affiliations:** 1 Psychology Department Leeds School of Social Sciences Leeds Beckett University Leeds United Kingdom

**Keywords:** eHealth literacy, irrational health beliefs, predictors, self-efficacy, teenagers and young adults, need for cognition, exploratory factor analysis

## Abstract

**Background:**

Increasingly, teenagers and young adults (TYAs) seek out health information online; however, it is not clear whether they possess electronic health (eHealth) literacy, defined as “the ability to select, appraise, and utilize good quality health information from the internet.” A number of factors are included in the Lily model proposed by Norman and Skinner underpinning the development of eHealth literacy. It is important to understand which elements may influence the development of eHealth literacy in young people, as the current generation will continue to “Google it” when faced with a health problem throughout their lives.

**Objective:**

The objectives of this study are to explore potential factors influencing young people’s eHealth literacy and explore the underlying constructs of the eHealth Literacy Scale (eHEALS) in a population of UK university students.

**Methods:**

A total of 188 undergraduate psychology students from a large UK University were recruited as an opportunity sample. Of these, 88.8% (167/188) of participants were female with a mean age of 20.13 (SD 2.16) years and the majority were White British (159/188, 84.6%). Employing a cross-sectional design TYAs completed the following measures exploring eHealth literacy (eHEALS): Irrational Health Belief Scale; Newest Vital Sign (NVS), a measure of functional health literacy; Need for Cognition Scale, a preference for effortful cognitive activity; and General Self-Efficacy (GSE) Scale, exploring personal agency and confidence. The eHEALS was also subject to exploratory factor analysis (EFA), for which in addition to the total variance explained, the scree plot, eigenvalues, and factor loadings were assessed to verify the structure.

**Results:**

eHEALS and GSE were significantly positively correlated (*r*=0.28, *P*<.001) and hierarchical linear modeling revealed GSE as the significant predictor of scores on the eHEALS (*F*_1,186_=16.16, *P*<.001, *R*^2^=0.08), accounting for 8.0% of the variance. Other notable relationships were GSE and need for cognition (NFC) were also positively correlated (*r*=0.33, *P*<.001), and NFC and irrational health beliefs were significantly negatively correlated (*r*=–.14, *P*=.03). Using Spearman correlations, GSE and NVS (*r*_s_=0.14, *P*=.04) and NFC and NVS (*r*_s_=0.19, *P*=.003) were positively correlated. An EFA revealed the scale to be stable and identified a 2-factor structure related to information acquisition and information application.

**Conclusions:**

This is the first study in the UK to explore relationships between these key variables and verify the structure of the eHEALS in a TYA population in the UK. The findings that self-efficacy has a major influence firmly consolidate its status as fundamental to the development of eHealth literacy. Future studies will explore the influence of body image and the development of eHealth literacy in more diverse TYA populations.

## Introduction

### Background

Almost all young adults in the UK are shown to be recent internet users [1] with teenagers present online for over 6 hours a day [2]. Indeed, most young adults consider the internet a valid source of health information [3,4] and compared with “digital immigrants,” “digital natives” (ie, those who have grown up in the digital age) seek out more health information electronically [5]. While the internet has transformed and widened access to health information, there has been concern about the quality of electronic health (eHealth) resources [6,7] and indeed whether people can select good quality health information on the internet. Undoubtedly, increasing the health literacy of populations is now seen as essential and is fast becoming a global challenge [8]. In England, 15% of adults are classed as functionally illiterate [9] and 32 million adults in the United States are unable to read [10]. However, successfully accessing eHealth resources goes beyond functional literacy skills and requires “eHealth literacy,” defined as the ability to “seek out, find, evaluate and appraise, integrate, and apply what is gained in electronic environments toward solving a health problem” ([11], p. 2).

### Young People and eHealth Literacy

Intuitively one would assume that young people as “digital natives” would be competent users of the technology they have been brought up with; however, this is not always the case [12]. Equally they are not always capable users of eHealth resources [13]. This lack of competency and fundamental concerns around the extent of misinformation in eHealth resources [14] have the potential to impact public health in terms of individual health outcomes and unnecessary burdens on health care systems [15]. Therefore, it is important to know whether young people can discern if the information gained from eHealth resources is of good quality and appropriate to their needs. To this end, Norman and Skinner [16] have developed a measure of perceived eHealth literacy—the “eHealth Literacy Scale” (eHEALS). Good reliability and validity from the eHEALS are demonstrated in young people in the United States [17] and the measure has been successfully translated into several languages [18-20]. Thus, if we are to successfully measure eHealth literacy, it is also important to understand factors that may influence the perceived ability of teenagers and young adults (TYAs) to successfully navigate internet-provided health resources. This is particularly crucial as this is the first generation to have grown up with access to eHealth resources and will continue to “Google it” when faced with a health problem throughout their lives. Therefore, it is important to gain a deeper understanding of what factors potentially influence eHealth literacy in young people. Norman and Skinner [11] propose that eHealth literacy is underpinned by a set of complex skills depicted by the 6-factor Lily model. The model proposes the following 6 overlapping literacies that determine and influence eHealth literacy: traditional literacy, health literacy, information literacy, scientific literacy, media literacy, and computer literacy. Here we explore putative variables which could enable the exploration of eHealth literacy and the 6-factor Lily model in young adults today.

### Potential Factors Influencing eHealth Literacy

#### Functional Health Literacy

One of the fundamental variables underpinning the Lily model is functional health literacy (FHL; ie, the literacy and numeracy skills required to read and understand health information). FHL is an essential skill in navigating health information and pivotal to successful engagement with health resources over the internet. Recent studies have shown that there is a relationship between FHL and eHealth literacy; higher scores in FHL were correlated with higher scores on the eHEALS in a sample of Italian adults [21]. Until recently we have had few reliable and valid ways to measure FHL in the UK. However, the Newest Vital Sign (NVS), a new measure of FHL, has recently been adapted [22] and validated against the Test of Functional Health Literacy in Adults (TOFHLA) in the UK [23], thus enabling an exploration of a potential relationship between FHL and eHealth literacy in the TYA population in the UK.

#### Need for Cognition

Norman and Skinner cite Feuerstein [24] who describes media literacy as successful navigation for those who develop metacognitive reflective strategies to enable them to reflect, reason, and discern media information. As such, media literacy has been adopted as an important component of the Lily model. Although not all those who use the media have the same level of cognitive facility, we often differ in our willingness to engage in cognitive activity to aid our understanding. Need for cognition (NFC) is an individual difference which can be described as “people’s tendency to engage in, and enjoy, effortful cognitive activity” ([25], p. 130). Those with high NFC enjoy problem solving, actively search for information, and reflect on the findings [26]. By contrast, those with low NFC avoid problem solving and rely on others to process and present information (eg, celebrities or powerful others). In terms of internet use, NFC is positively correlated with all internet activities involving cognitive thought [27], and also linked to cancer screening uptake such that matching health messages to information-processing styles improved mammography attendance at 6 months in high NFC women [28]. More recently NFC was explored in relation to eHealth literacy in US university students, establishing a positive relationship between high NFC and eHEALS scores, and showing those with high NFC scores would be more likely to seek out health information online [29]. Thus, it seems a natural progression to explore whether this relationship between NFC and eHEALS exists in a UK university sample.

#### Irrational Health Beliefs

However, it is not only cognitive capacity which underpins success in processing and utilizing health information. Generally, it is assumed that health and scientific information are processed rationally. However, some (particularly people with anxiety) have “irrational health beliefs” (IHBs) which can be explained by the cognitive–behavioral model of hypochondriasis [30,31]. People with IHBs believe serious illness to be more prevalent than it is, believe fast action health input is imperative, and fear disastrous health outcomes (if not treated) [32]. This irrationality can have a dramatic influence on young peoples’ information literacy and their use and interpretation of health information and decision making (eg, in not adequately responding to the real costs and benefits of alcohol consumption) [33]. Certainly, it was found that people with higher IHBs have poorer health and are less likely to adhere to treatments [34,35]. Clearly, this could be problematic for seeking health information via the internet and using this appropriately. Indeed, this “worried and wired” combination has recently been shown to be detrimental where individual levels of health anxiety have negatively influenced the relationship between online health information seeking and health care utilization [36]. Thus, in terms of influencing the health, science, and media literacy of young people, IHB is a concept worthy of study.

#### Self-Efficacy

Underpinning the previously mentioned components of the Lily model, Norman and Skinner state that electronic health literacy is born out of social cognitive theory proposed by Bandura [37], with self-efficacy (SE) being a major component [38]. SE is defined as “beliefs in one’s capabilities to mobilize the motivation, cognitive resources, and courses of action needed to meet given situational demands” ([39], p. 408). High SE [40] is related to engaging and maintaining healthy behaviors, and is a key component of most major health models [41]. SE is viewed as the factor to bridge the intention–behavior gap, the most eagerly pursued link of health behavior change. SE is pivotal to seeking out information and acting appropriately on it via the internet and is likely to improve the likelihood of utilizing the resources successfully [42]. Therefore, measuring young people’s general levels of self-efficacy (GSE) is an important factor to explore in relation to perceived eHealth literacy.

The putative factors we have reviewed here all relate to young people’s experiences of help seeking via the internet and consequent health behavior. Further, these factors reflect the overlapping literacies of the Lily model proposed by Norman and Skinner [11], namely, health literacy, information literacy, scientific literacy, media literacy, and computer literacy.

With regard to the scale to measure eHealth literacy (eHEALS) [16], this been evaluated with young adults and baby boomers in the United States, whereas in the UK and New Zealand this has been evaluated only in the latter [43]. Therefore, it would be prudent to also conduct an exploratory construct analysis on the eHEALS in a UK TYA population.

### Aims

The primary aim was to explore whether factors potentially underpinning the 6 facets of the Lily model [11] (including NVS [FHL], IHBs, NFC, and GSE) are associated with and are significant predictors of eHealth literacy in young people measured by the eHEALS [16].

A secondary aim was to explore the underlying constructs of the eHEALS using an exploratory factor analysis (EFA) in a sample of university students in the UK.

## Methods

### Participants

Participants (n=188) were undergraduate psychology students from a UK University recruited via opportunity sampling in research methods sessions. Of these, 167 participants were female (88.8%), with a mean age of 20.13 (SD 2.16) years. The majority of participants identified their ethnicity as White British 159/188 (84.6%) and said that English was the main language spoken at home (181/188, 96.3%). Other participants described their ethnicity as Mixed 4/188 (2.1%); Pakistani, Indian, or Asian 13/188 (6.9%); any other White background 2/188 (1.1%); or Black 2/188 (1.1%). The majority were unmarried 182/188 (96.8%) and living in shared accommodation 122/188 (64.9%). Most participants 139/188 (73.9%) had not seen a health professional in the past 2 weeks. Of those that had, most had seen a general practitioner (family doctor; n=26) or a physiotherapist (n=4).

### Materials

#### eHealth Literacy Scale (eHEALS)

The eHEALS [16] is an 8-item measure of eHealth literacy, and has an internal consistency (Cronbach α) of .88. An example question is “I know **how to use** the Internet to answer my questions about health.” The responses range from 1 (strongly disagree) to 5 (strongly agree), with higher scores indicating a higher level of eHealth literacy.

#### Newest Vital Sign (NVS)

The NVS [22,23] is a measure of FHL, and has an internal consistency (Cronbach α) of .70. It includes a maximum of 7 questions, in which participants answer questions (involving calculations) relating to the nutritional information label on a tub of ice cream. The responses to questions are scored as either correct or incorrect and range from 0 to 6, with higher scores indicating higher levels of FHL.

#### Need for Cognition Scale

The Need for Cognition Scale [44] is an 18-item measure which assesses a tendency to enjoy and engage in effortful cognitive activity. It has an internal consistency (Cronbach α) of .88, suggesting good reliability. An example question is “I really enjoy a task that involves coming up with new solutions to problems”. Items are scored from –4 (very strong disagreement) to 4 (very strong agreement), with higher scores signaling greater NFC.

#### Irrational Health Belief Scale

The Irrational Health Belief Scale [45] is a 20-item scale assessing the tendency to appraise health-related information in an irrational manner, and has an internal consistency (Cronbach α) of .90. It includes a series of vignettes to which participants have to rate their perceived response from a 5-point scale ranging from 1 (Not at all like I would think) to 5 (Almost exactly like I would think). For example, “Your doctor recommends a new medication for an ongoing health problem and indicates that about 10% of patients experience unpleasant side effects from the medicine. You think to yourself, ‘If anyone is going to have side effects, it’s going to be me’.” Scores are summed, with higher scores relating to higher IHBs.

#### General Self-Efficacy (GSE) Scale

The General Self-Efficacy (GSE) [46] scale is a 10-item measure, which captures the extent to which individuals are optimistic about their ability to cope with challenging situations, and has an internal consistency (Cronbach α) of .90. An example question is *“*I can always manage to solve difficult problems if I try hard enough.” The measure has Likert scale scoring, with responses ranging from 1 (not at all true) to 4 (exactly true). The answers are summed, with total scores ranging from 10 to 40. A higher score indicates greater SE.

### Procedure

Ethical approval from the Leeds Beckett Psychology Ethics Committee was granted on December 12, 2017 (number: PH/AW/121217). Participants took part in this study in order to generate data for them to analyze in a research methods module, and gave informed consent for these data to be used in further research. All measures were programmed into Qualtrics, a web-based survey tool, and participants completed these in-exam conditions during research methods sessions over the course of 1 week in March 2018.

### Data Analysis

All data analyses were conducted using IBM SPSS Statistics (version 26). Internal consistency of the scales was examined using the Cronbach alpha coefficient. The psychometric properties of the eHEALS in a UK sample were explored using EFA. We initially examined correlations between all key variables. We had theoretical grounds to suggest that GSE would be a significant predictor of eHealth literacy, therefore a hierarchical multiple regression was performed by entering GSE first and then examining the impact of NFC, IHBs, NVS, and GSE on the outcome variable eHEALS. For the NVS, 18% of the data were missing, therefore mean scores were inputted; missing data were <0.01% for other variables.

Data assumption tests were performed prior to conducting the linear regression, including Cook distance, collinearity, variance inflation factor, Durbin–Watson, and homoscedasticity. To test for normality, skewness and kurtosis values were computed prior to correlation analysis.

An EFA was used to examine the eHEALS structural validity on the 8 data item set (N=188 cases). The minimum recommended sample size to conduct an EFA is 100 [47] and if factors emerge with 4 or more loadings over 0.6, then this would be deemed reliable regardless of sample size [48]. In addition to the total variance explained, the scree plot, eigenvalues, and component loadings were assessed to verify the factor structure of the eHEALS in this population.

## Results

Descriptive statistics for all key variables are presented in Table 1. All data met the requirements for parametric testing, with skewness and kurtosis values between +2.0 and –2.0 [49], with the exception of the NVS which was negatively skewed (as expected in an educated undergraduate sample). After a verbal consultation with a colleague (G. Rowlands, London South Bank University, personal communication), the decision was made not to transform the NVS data, as this has not been done using this measure before. Therefore, a Spearman nonparametric correlation coefficient (ρ) was employed for the NVS, thus elsewhere the NVS results should be interpreted with caution.

**Table 1:** Descriptive statistics and internal consistency for key variables.

Measure	N	Range	Mean (SD)	Cronbach α
Electronic Health Literacy Scale	188	12-40	29.46 (4.91)	.84
Newest Vital Sign	154	2-6	5.44 (0.94)	.60
General Self-Efficacy Scale	188	20-40	29.78 (3.84)	.80
Irrational Health Belief Scale	188	18-64	31.30 (9.12)	.85
Need for Cognition Scale	188	23-85	7.02 (18.97)	.90

Correlations between key variables are presented in Table 2. The main finding of interest is the significant positive correlation between eHEALS and GSE (*r*=0.28, *P*<.001). No other significant relationships were noted between eHEALS and other key variables. The GSE and NFC scores were also positively correlated (*r*=0.33, *P*<.001), and NFC and IHBs were significantly negatively correlated (*r*=–0.14, *P*=.03). Using Spearman correlations, GSE and NVS (*r*_s_=0.14, *P*=.04) and NFC and NVS (*r*_s_=0.19, *P=*.003) were positively correlated.

**Table 2:** Correlation coefficients for key variables^a^.

Measure	1	2	3	4	5
1. Electronic Health Literacy Scale	—^b^	0.02	0.28^c^	–0.11	0.11
2. Newest Vital Sign	0.07	—	0.14	–0.08	0.20^c^
3. General Self-Efficacy Scale	0.28^c^	0.80	—	–0.14^d^	0.33^c^
4. Irrational Health Belief Scale	–0.11	–0.18^d^	–0.14	—	–0.14^d^
5. Need for Cognition Scale	0.11	0.19^c^	0.33^c^	–0.14^d^	—

^a^All correlations are Pearson with the exception of NVS where Spearman was performed.

^b^Not applicable.

^c^Correlation is significant at the 0.01 level.

^d^Correlation is significant at the 0.05 level.

All assumptions of the regression analysis were met, and a hierarchical procedure was performed to assess if the variables GSE, NVS, IHB, and NFC could predict eHealth literacy measured by the eHEALS. GSE was entered first into the model (Model 1) and this explained a significant proportion (8.0%) of the variance in eHealth literacy (*F*_1,186_=16.16, *P*<.001, *R*^2^=0.08). Model 2 (including GSE, NFC, IHB, and NVS) explained a nonsignificant 0.6% increase in the variance (*F*_4,183_=4.30, *P*=.80, *R*^2^=0.086). Together, both models explained 8.6% of the total variance. Table 3 shows that only GSE was a significant predictor in each model. The internal consistency of most scales was good (ie, ≤0.80), excluding the NVS, for which this was 0.60.

**Table 3:** Beta coefficients, standard errors (SEs), standardized betas, and significance value for each model and predictors therein.

Factors		β coefficient	SE β	Standardized β	*P*
Constant		18.71	2.70	—^a^	<.001
**General self-efficacy (Model 1)**		0.36	0.90	.28	<.001^b^
	Constant	20.84	3.71	—	<.001
**General self-efficacy (Model 2)**		0.34	0.09	.27	<.001^b^
	Need for Cognition	0.00	0.02	.00	.93
	Irrational Health Beliefs	–0.04	0.03	–.08	.28
	Newest Vital Sign	–0.07	0.32	–.01	.83

^a^Not applicable.

^b^Significant at the 0.01 level.

Using principal axis factoring an EFA was conducted on the 8 items with varimax rotation. The Kaiser–Meyer–Olkin measure verified sampling adequacy at 0.80 above the minimum criterion of 0.50, and all Kaiser–Meyer–Olkin values for individual items were 0.65 or more. The Bartlett test of sphericity was also significant at *P*<.001. All items on the eHEALS correlated significantly at *P*=.001. A determinant value of 0.023, which is greater than the required value (ie, >0.00001), revealing collinearity levels, was not detrimental to the analysis, and thus, no items were removed.

The eHEALS performed well in terms of psychometrics in this sample (Table 4): we calculated a Cronbach α value of .84 for the total eHEALS score, whereas for factors 1 and 2 this was .90 and .77, respectively, but when removing item 8, which had a coefficient of less than 4, the Cronbach α for factor 2 increased to .80. However, almost one-third scored the maximum on the eHEALS overall (Table 4).

**Table 4:** Descriptive statistics, floor, and ceiling effects for eHEALS^a^ overall and its factors 1 and 2.

Measure		Mean (SD)		Floor effect (% of min score)		Ceiling effects (% max score)	
**eHEALS**		29.46 (4.91)		16.48		28.12	
	Factor 1: Information acquisition		11.46 (0.80)		2.65		10.11
	Factor 2: Information application		19.07 (1.00)		13.82		17.70

^a^eHEALS: Electronic Health Literacy Scale.

The scree plot confirmed that two factors had Eigen values over the Kaiser criterion of 1 and together explained 64.6% of the variance. Table 5 shows the results of the EFA, suggesting that factor 1 (items 1-3) represents information acquisition and factor 2 (4-8) information application.

**Table 5:** Summary of the exploratory factor analysis on the eHEALS^a^.

Items on the eHEALS	Factor 1 (acquisition)	Factor 2 (application)
1. I know where to find helpful health resources on the Internet	.91	
2. I know how to find helpful health resources on the Internet	.78	
3. I know what health resources are available on the Internet	.72	
4. I know how to use the health information I find on the Internet to help me		.74
5. I know how to use the Internet to answer my questions about health		.63
6. I feel confident in using information from the Internet to make health decisions		.63
7. I have the skills I need to evaluate the health resources I find on the Internet		.62
8. I can tell high quality health resources from low quality health resources on the Internet		.32
Eigen values	4.0	1.2
Percentage of variance	49.4	15.1

^a^eHEALS: Electronic Health Literacy Scale.

## Discussion

### Principal Findings

In this exploratory study we have been the first to investigate relationships and potential predictors of key variables influencing eHealth literacy sample of UK University students. A highly significant positive relationship was found between eHEALS and GSE scores, but eHealth literacy did not significantly correlate with any other factor. However, significant positive correlations between NFC and the NVS, and between NFC and GSE were demonstrated as well as a significant negative association between NFC and IHB. Our secondary aim was to explore the underlying construct of the eHEALS in a UK TYA sample using EFA, where we found 2 underlying factors within the scale related to information *acquisition* and information *application*.

Despite the expected negatively skewed distribution indicating higher scores on the NVS in our student sample, FHL measured by the NVS was not found to be a significant predictor of eHealth literacy. Previously Del Giudice and colleagues [21] demonstrated large associations between functional and eHealth literacy; however, in their study, FHL was not directly measured but rather assumed (as a proxy measure) based on studying or working in the health sector (eg, physicians, nurses, and allied health professions). Further, Del Giudice et al [21] found that for older and better educated participants eHEALS scores were higher. Our sample comprised psychology students (a health-focused subject), but perhaps the younger age of our sample (mean age 20) could have been an influencing factor in the lack of significant association with the eHEALS scores.

In line with the literature, those who scored highly in our sample on the NVS (a test of functional literacy) also scored highly on the NFC scale. This suggests an overlap between these 2 variables in that someone who prefers effortful cognitive activity would be more likely to score highly on a functional literacy test [26]. Our findings do not support the health literacy aspect of the Lily model in terms of demonstrating a relationship between functional and eHealth literacy. However, the expected negatively skewed scores in this educated sample and the internal consistency of .60 of this scale would suggest proceeding with caution in interpretation of our findings. In future studies it may be useful to explore FHL with the NVS in a more diverse population of young people who are either unemployed or who have not had a university education, as there is an urgent need to explore the factors driving FHL in *all* young people [50] to enable successful health promotion strategies.

The significant negative association between NFC and IHBs was as predicted, as IHBs are usually strongly held and people displaying IHBs have a limited need or desire to acquire new information to challenge or inform these beliefs [32]. Although counterintuitively, this did not transfer into a tangible negative association with eHealth literacy.

### Self-Efficacy

SE has been shown to be a pivotal component in the adoption and execution of healthy behaviors [51], and that it being a significant predictor of eHealth literacy would seem entirely plausible. Previously, internet SE was deemed to play a key role in the process of using the internet to acquire health information [52]. With this in mind it may be useful to explore the relationship between eHealth literacy and a measure of health-related SE (eg, [53]). Using a dedicated health SE measure may have accounted for more variance in the model and therefore may be a greater predictor. Interestingly, others have argued that the eHEALS actually measures SE rather than eHealth literacy [29]; indeed, the question prefixes (eg, “I know how to” and “I have the skills to”) would seem to tap into confidence and SE rather than assess performance. Perhaps, implementation of internet skills could not be predicted from high eHEALS scores, suggesting an incongruence between perceived and actual eHealth literacy [54]. We echo the thoughts of Britt and Hatten [29] who call for the relationship between SE and eHealth literacy to be fully explored.

### Need for Cognition

We found a lack of significant correlation and predictive impact of NFC on the outcome variable (eHEALS) in contrast to the US study of young university students in which an association between the 2 variables was demonstrated [29]. It is useful to try to explain the reasons for failure to replicate these findings in a UK sample. The mean eHEALS scores and standard deviations were very similar in this study and the one conducted in the United States [29], and both samples were of a similar age. However, the US study had a larger sample yielding greater power to detect significant associations. Moreover, our UK sample produced higher NFC scores than participants in the US study, but the latter did not provide information on the types of courses the students were enrolled in. Conceivably, the type of course influenced the results, as Del Giudice and colleagues [21] found that participants’ exposure to health-related study was associated with higher eHealth literacy scores.

### Scale Structure

Previously the eHEALS was thought to be a unidimensional scale [16,17]; however, in the UK a confirmatory factor analysis and structured equation modeling in a large study with baby boomers in the United States, UK, and New Zealand (born between 1946 and 1954) found the eHEALS to have 3 distinct scales related to the Lily model [43]. They found items 1 and 2 related to awareness, items 3-5 related to skills to access resources, and items 6-8 evaluation of resources (self-efficacy). The authors recognize that more work needs to be done, particularly in other age groups to verify the 3-factor structure. Our findings that the eHEALS related to 2 distinct constructs, namely, information *acquisition* (items 1-3) and information *application* (items 4-8), concur with those of Soellner et al [55], who found the 2-factor structure to be more compelling than a single one. However, we would recommend continuing to explore the construct validity of the scale in different populations.

### Limitations

This was a cross-sectional design and as such only measured eHealth literacy at one time point, although it may be more revealing to measure this longitudinally to explore temporal changes and responses to changing circumstance (eg, an individual’s own health concerns). The skewed distribution of the NVS data and low internal consistency ensure that we proceed with caution and thus cannot conclusively state that FHL does not influence eHealth literacy in a UK TYA population. However, we also must question “Does perceived eHealth literacy (as measured by the eHEALS) translate into competent performance on the internet to gain health information?” Others have also found that the expected strong positive relationships between eHEALS scores and internet performance were absent [53]. This suggests that future studies should also focus on measuring practical internet tasks along with perceived eHealth literacy as a comprehensive measurement of true eHealth literacy.

In this study, because GSE only accounted for a small proportion of variance in our predictive model, we must speculate that alternative, more potent predictors of eHealth literacy exist. Gilstad [56] has proposed further aspects to eHealth literacy, thereby expanding the Lily model proposed by Norman and Skinner [11]. Four additional aspects to the Lily model were proposed: bodily experience, procedural literacy, contextual and cultural literacy, and communicative expertise. These additional factors are particularly pertinent to young people as they navigate seeking health information online, particularly knowledge of the norms, values, rules, and regulations in social situations (contextual and cultural literacy) and bodily experience (the ability to identify a health problem in one’s own body). A recent work [57] demonstrated that delayed help seeking for potential breast cancer in females was associated with dissatisfaction with their breasts. Given that body dissatisfaction increases for both males and females as they transition to young adulthood [58], it would be interesting to explore whether body dissatisfaction could impact on their eHealth literacy.

### Conclusion

We are reassured that NHS England [59] is working toward improving health literacy to reduce health inequalities, and perhaps there should be a greater directive to expand this initiative to include eHealth literacy. eHealth literacy should not be seen as a static state, but rather as a dynamic evolving skill set that will develop over time and in response to individual need and circumstance [11]. This would seem a logical step forward to serve digitally native young people as these are the generation that will “Google it” for health information into adulthood.

In conclusion, this is the first study in the UK to explore relationships between these key variables in a TYA population, and to perform EFA on the eHEALS with a TYA sample in the UK. As such, we can confirm the stability of the scale. The findings that SE has a major influence on eHealth literacy should consolidate its status as underpinning the Lily model, and as a fundamental starting point from where eHealth literacy is developed and understood. Future studies will explore physical and mental health status, health SE, and body image as potential predictors of eHealth literacy in more diverse TYA populations.

## Abbreviations

EFA: exploratory factor analysis
eHEALS: Electronic Health Literacy Scale
FHL: functional health literacy
GSE: general self-efficacy
IHB: irrational health belief
NFC: need for cognition
NVS: Newest Vital Sign
SE: self-efficacy
TOFHLA: Test of Functional Health Literacy in Adults
TYAs: teenagers and young adults
